# Supplementary material for: Content-rich biological network constructed by mining PubMed abstracts
Source: BMC Bioinformatics. 2004 Oct 8;5:147. doi: 10.1186/1471-2105-5-147 (PMC528731; doi:10.1186/1471-2105-5-147)
Supplement: Additional File 2 — The original results of the above study (non-essential files are deleted to keep the file size under the limit set by BMC bioinformatics). [file 1471-2105-5-147-S2.bz2 › chilibotAdditionalFile2/dip05/384ID1375038E9797/html/NTRK2_BDNF.html]

 


 **NTRK2** and **BDNF** 
  
Found 857 abstracts in PubMed, retrieved 05.  
 

 What does Google say? 
 PDF only 
| .edu only 

---

**Interactive relationship** (e.g. stimulation, inhibition, etc)

**Neutral relationship**- **BDNF**  then binds to and activates its cognate receptor, receptor tyrosine kinase B TrkB  [ **NTRK2** ] .  Ref: 12853306 Ann N Y Acad Sci< MedlineTA>Ann N Y Acad Sci,

**Non-interactive relationship** (e.g. studied together, co-existance, homology, etc.)

- As the VMN undergoes sexual differentiation, we compared  **BDNF** , NGF, NT 3, and trkB  [ **NTRK2** ]  mRNA expression in the VMN in males and females at embryonic day 20 and PND 4, but found no differences between them.  Ref: 12824057 Brain Res Mol Brain Res, 2003
- Western blot analysis confirmed these results, showing that the lacrimal gland expresses NGF,  **BDNF** , NT3, and NT4 as well as the NTrs TrkA, TrkB  [ **NTRK2** ] , and TrkC and the p75 protein.  Ref: 12882781 Invest Ophthalmol Vis Sci, 2003
- RT PCR showed positive bands at the appropriate sizes for NGF,  **BDNF** , NT3, and NT4, and for the receptors TrkA, TrkB  [ **NTRK2** ] , TrkC, and p75.  Ref: 12882781 Invest Ophthalmol Vis Sci, 2003
- To determine which of the neurotrophins NTs nerve growth factor NGF, brain derived neurotrophic factor  [ **BDNF** ]   **BDNF** , neurotrophin 3 NT3, and neurotrophin 4 5 NT4 and their receptors NTrs, TrkA, TrkB  [ **NTRK2** ] , TrkC, and p75, are present in the adult rat lacrimal gland.  Ref: 12882781 Invest Ophthalmol Vis Sci, 2003
- Messenger RNAs encoding other neurotrophins, including nerve growth factor NGF and neurotrophin 3 NT 3 and the  **BDNF**  receptor trkB  [ **NTRK2** ] , were not selectively localized in neonatal VMN.  Ref: 12824057 Brain Res Mol Brain Res, 2003
- To test this hypothesis, we compared the ability of LIGA20 and  **BDNF**  to activate TrkB  [ **NTRK2** ] .  Ref: 12829403 Neurotox Res, 2002
- Cultured cortical progenitors express  **BDNF**  and NT 3, as well as their receptors TrkB tyrosine kinase  [ **NTRK2** ]  receptor B and TrkC.  Ref: 12832539 J Neurosci, 2003
